# Fertility intentions and influencing factors among women of reproductive age in Madhesh Province, Nepal: a population-based analysis of the 2022 Nepal demographic health survey

**DOI:** 10.1186/s12978-025-02228-2

**Published:** 2025-12-26

**Authors:** Bijaya Mani Devkota, Suman Chandra Gurung, Amshu Dhakal

**Affiliations:** 1 Central Department of Population Studies (CDPS), Tribhuvan University, Kathmandu, Nepal; 2 Manmohan Memorial Institute of Health Sciences (MMIHS), Kathmandu, Nepal

**Keywords:** Fertility intention, Woman of Reproductive Age, Nepal

## Abstract

**Background:**

Fertility intentions or desires for future childbearing are necessary variables in understanding reproductive behavior, particularly in transitioning societies like Nepal. Despite a national decline in fertility rates, regional disparities persist, notably in Madhesh Province, where socio-economic and cultural barriers remain entrenched. This study explores the socio-economic and demographic factors influencing fertility intentions among women of reproductive-age in Madhesh Province.

**Methods:**

A cross-sectional analysis of secondary data from Nepal Demographic Health Survey (NDHS) 2022 was conducted. This study concentrated on women aged 15–49 years and residing in Madhesh, selected through a multistage stratified sampling approach. A weighted sub-sample of 1,643 women was analyzed using descriptive statistics and binary logistic regression to explore the determinants of fertility intentions.

**Results:**

Age emerged as the strongest predictor: younger women (15–24 years) exhibited significantly higher fertility intentions than older cohorts. Parity showed a significant negative association with fertility intentions, with higher parity linked to lower desire for more children. Rural women had 33% lower odds of desiring more children compared to urban women. Initial associations with wealth, education, and caste/ethnicity were not significant after adjustment, while media exposure (especially newspapers) inversely correlated with fertility desires. Occupation, religion, and partner's education showed negligible effects.

**Conclusion:**

Fertility intentions in Madhesh Province are predominantly shaped by age, parity, rural–urban disparities, and media exposure. Interventions should prioritize the dissemination of reproductive health information through print media and the development of youth-friendly, rural-sensitive family planning programs.

## Plain Language Summary

This study explores the why women in Madhesh Province, Nepal, decide whether or not they want to have more children. Even though Nepal’s national fertility rate is declining, some areas like Madhesh still shows higher fertility in comparison to other provinces. Understanding why women want more or fewer children can help improve reproductive health services and provincial-level planning.

Using data from the Nepal Demographic Health Survey 2022, the study analyzed responses from women aged 1-49 living in Madhesh province. Factors such as age, education, residence (rural or urban), parity (number of living children) and media exposure were examined to see how they influence fertility intentions.

The results showed that younger women and those with fewer children were more likely to want more children, while rural women and those exposed to print media (like newspaper) were less likely. Fertility intention declined with increasing number of children, being highest among women with 1–2 children and lowest among those with five or more. These findings highlight the need for youth-centered and rural targeted reproductive health programs and for using accessible media to promote informed family planning choices in Madhesh Province.

These results suggest that health programs in Madhesh should focus more on young women and rural communities. Also, using print media like newspapers to share information about family planning could help more women make informed decisions about their reproductive health.

## Background

Fertility Intention can be defined as people’s desire to have children and pursuit of childbirth which is impacted by the expectation of the number, timing, gender and quality of the children [1]. It is an individual’s stated plan or decision regarding whether to have pr not have more children in future. Although fertility intention is widely used in population and reproductive-health research, it is also repeatedly confused with other similar concepts such as fertility preference, fertility desire, and fertility expectation. Although related, these concepts measure different dimensions of reproductive choice. Various chapters of DHS Program gave several definitions. Fertility preference refers to a person's wanted number or childbearing timing, reflecting normative aspirations or cultural ideals. Fertility desire reflects an individual's personal want or emotional tendency to have children, which is typically driven by affective and psychosocial considerations. Fertility expectation, on the other hand, refers to what people expect to happen considering their socio-economic situation and limitations.

For this research, fertility intention is defined as a proximal and implementable choice how frequently a woman reports planning to have (or not have) more children under the existing conditions in her life. Understanding fertility intentions is crucial for analyzing reproductive behavior, contraceptive use and population change, particularly in societies undergoing rapid demographic transitions such as Nepal. Understanding fertility intention is crucial for analyzing population trends, family planning behaviors and reproductive health dynamics. Unlike fertility outcomes, fertility intention reflects personal and household aspirations shaped by the socio-economic, cultural, and psychological factors [2, 3]. In Nepal, a country experiencing significant socio-political change, where women navigate tensions between tradition and modernity, investigating fertility intentions is especially relevant. These insights are vital for designing programs that promote reproductive autonomy, reduce unwanted pregnancies, and improve maternal and child healthcare [4, 5]. This study addresses the need for a clear conceptualization of fertility intention, defined here as an individual’s expressed desire or plan to have children in the future.

Nepal’s total fertility rate (TFR) declined from 4.6 in 1996 to 2.1 in 2022 [6, 7]. However, provincial disparities persist. The Provincial fertility estimates and socio-economic indicators of NDHS 2022 and CBS 2021 data demonstrated that Madhesh has one of the highest total fertility rates (2.9), lowest female literacy (55%), and lowest modern contraceptive prevalence (34%) among provinces of Nepal. The fertility intentions of women in this region remain poorly understood, especially amidst recent federalization and evolving health policies. These contextual factors make Madhesh a critical region for examining fertility behavior in Nepal’s federal system.

Fertility intentions are shaped by a complex set of demographics, socio-economic and cultural factors including age, parity, education, household income, employment, access to services and exposure to media [8, 9]. Empowerment through education or decision-making autonomy typically reduces desired family size and increases contraceptive use [10]. Conversely, poverty, gender discrimination, and limited contraceptive access create structural restrictions, particularly in the regions like South Asia and sub-Saharan Africa, hindering women’s ability to align intentions with outcomes [11, 12]. In Nepal, however, women’s fertility intentions are often constrained by the expectations of husbands and in-laws, leaving little room for personal negotiation. [13, 14].

While national-level studies in Nepal reported education, wealth and age as major predictors, Women here face layered disadvantages regarding reproductive choice [15]. For instance, Terai Dalit and Muslim women in Madhesh experience severely limited access to antenatal care and skilled birth attendance, yet their fertility aspirations remain under-researched [16]. Although national studies identify education, age, and wealth as key determinants, these findings may not fully capture the Madhesh Province’s unique socio-cultural environment, where deep-rooted patriarchy persists [17]. Understanding the interplay between these factors and fertility intentions is critical for addressing regional disparities in family planning and reproductive health.

Previous evidence from culturally similar regions of India and rural Ethiopia suggests that fertility intentions are strongly mediated by local norms, dowry practices and gender dynamics [18, 19]. Such findings highlight the need for localized research to plan and design interventions suited to provincial realities rather than relying solely on national average data.

Guided by the demographic and health transition framework, this study examines the determinants of fertility intentions among women of reproductive age in Madhesh province using the most recent NDHS 2022 data. By incorporating key variables such as parity, age, residence, education, caste and media exposure, it offers new insights into factors shaping women’s reproductive plans in a province where fertility decline has been slower than elsewhere in Nepal. Findings are expected to inform provincial level reproductive health strategies and contribute to the broader evidence base on fertility behavior in transitioning societies.

The demographic and health transition framework provides a useful lens to understand fertility intention shifts in Nepal. While rising literacy, health infrastructure, and urbanization generally predict declining fertility preference, this decline often conflicts with persistent social norms and economic structures. In Madhesh, for instance, fertility intentions remain high alongside an unmet need for contraception [20, 21]. Bridging this gap requires timely, geographically disaggregated empirical evidence.

This study utilizes the data from the Nepal Demographic Health Survey (NDHS) 2022 to examine fertility intentions and their socio-economic and demographic determinants among women aged 15–49 in Madhesh Province. Using logistic regression within a population-based framework, it identifies key predictors of fertility intentions such as education, household wealth, occupation, age, parity, caste/ethnicity, and media exposure. Provincial-level analysis is particularly important in Nepal’s post-federalization context, where provincial governments are now responsible for health planning and service delivery [22] Despite increasing national attention to fertility trends, existing studies often rely in aggregated national data and overlook subnational diversity of the country. Empirical research specifically focused on Madhesh province, a region marked by pronounced socio-economic inequalities, embedded gender norms and cultural heterogeneity remains limited [23].

This study addresses that gap by providing a disaggregated, evidence-based understanding of fertility intentions in Madhesh province. The findings have both academic and policy significance offering insights to inform culturally sensitive reproductive health programs, guide resource allocation and support the empowerment of marginalized women [24]. Ultimately, this research contributes to linking reproductive intentions with outcomes in one of the Nepal’s most socio-economically vulnerable regions.

## Methods

This study employed a quantitative, cross-sectional design using secondary data from the 2022 NDHS Survey to assess factors influencing fertility among reproductive-age women in Madhesh Province.

### Study design

The analysis used a nationally representative dataset from NDHS [25], collected via a multi-stage stratified cluster sampling method. The NDHS gathered demographic, health, and reproductive information from women aged 15–49 years across all the provinces of Nepal. This study particularly analyzed data for women residing in Madhesh Province. The cross-sectional approach allowed for the examination of the relationship between socio-demographic factors and fertility intentions at a single point in time.

### Sample and sampling procedure

The NDHS used a two-stage stratified sampling design. The initial selection involved rural and urban strata, followed by the systematic selection of enumeration areas (EAs) and then households within those EAs. For this study, a weighted sample of reproductive-age women (15–49 years) living in Madhesh Province was extracted. Women who provided responses to key fertility preference questions regarding desire for more children, timing, and family size were included in the analysis. The final sample size was determined based on completeness of responses and the availability of relevant variables for analysis.

#### Model of diagnostics sample frame

**Fig. 1:**
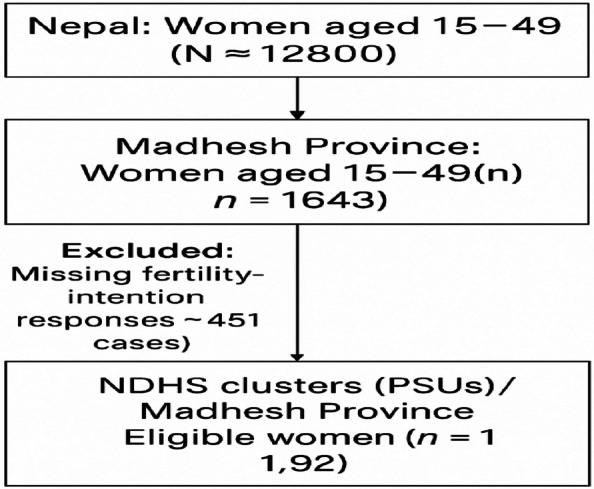
Model of Diagnostics sample frame. Source for Fig. 1: Computed from NDHS 2022

Figure 1 presents the Diagnostic Sample Frame Model showing the hierarchical sampling plan and derivation of the analytical sample of the total population of reproductive age. The sampling was based on NDHS clusters (PSUs) and then household of the eligible women respondents (1192 from 12,800 national data) were selected with the use of PPS techniques and design weights.

### Data analysis methods

Descriptive statistics characterized the respondents background characteristics. Logistic regression analyses identified the socio-demographic and reproductive health factors associated with fertility intentions. Both unadjusted and adjusted odds ratios (ORs) with a 95% confidence interval (CIs) were calculated. Statistical significance was set as *p* < 0.05.

An applied NDHS sampling weights, PSU, and strata. The survey-weighted logistic regression yielded a highly significant model (Wald χ^2^ = 182.4, *p* < 0.001) with a pseudo-R^2^ of 0.29. Variance Inflation Factors (VIF)indicated low inter-predictor correlation (max VIF = 2.1). The sparse-cell issues were resolved through category merges, and sensitivity checks using a simplified model confirmed the robustness of the main results (Table 1).

**Table 1:** Model-diagnostic weighted logistic regression

Diagnostic item	Description/Value
Survey design	svyset psu = v021; strata = v022; pweight = v005/1 000 000
Estimator used	Survey-weighted logistic regression (svy: logit)
Sample size (unweighted)	1 643 (observed); 1192 (Adjusted model)
Wald χ^2^ (df = 12)	182.4 (*p* < 0.001) — Model significant
Pseudo R^2^	0.29
Multicollinearity (VIF)	Mean = 1.6; Max = 2.1 → no multi-collinearity
Zero/near-zero cells	Resolved by merging sparse age (40–49) and minor religion groups
Survey weights applied	NDHS design accounted for clustering and stratification
Robustness check	Simplified model (excluding media variables) → same direction and significance
Interpretation	Findings stable; model fit adequate; no multi-collinearity or design bias detected

The main result measure was fertility intention, derived from NDHS questions v602 and v605, which ask a woman how many more children she wants. It was coded dichotomously as: code 1 = desires more children, and 0 = does not desire more children, including undecided, sterilized, or infecund respondents. This definition meets DHS analytical requirements and allows for comparability with other fertility intention studies [6, 26]. Explanatory variables of major relevance were chosen on the basis of theoretical and empirical evidence supporting their association with fertility behavior (Table 2).

**Table 2:** Description of variables

Variable	NDHS Code	Recoding	Reference
Dependent Variable	v602/v605	1 = Wants more children; 0 = Does not want/undecided/sterilized	—
Age (years)	v012	15–19, 20–24, 25–29, 30–34, 35–39, 40–49	15–19
Residence	v025	Urban/Rural	Urban
Parity (number of living children)	v218	0, 1–2, 3–4, 5 + (children alive)	0
Education (women)	v106	No education/Primary/Secondary/Higher	Higher
Newspaper exposure	v157	Not at all/< 1 per week/≥ 1 per week	≥ 1 per week
Wealth quintile	v190	Poorest → Richest (5-level DHS index)	Richest
Religion	v130	Hindu/Muslim/Other (merged Buddhist, Kirat, Christian)	Hindu
Caste/Ethnicity	v131	Other Terai/Terai Dalit/Other	Hill Brahmin

### Ethical consideration

This study used publicly available secondary data from the 2022 NDHS, which was conducted by the Ministry of Health and Population (MoHP) in collaboration with ICF, following all ethical guidelines. The data protocol ensures participant confidentiality and safety. No personal identifiable information was used in this analysis, and data access was granted by the DHS Program [7].

## Results

Analysis of the 2022 NDHS data revealed multiple factors associated with fertility intentions among women in Madhesh Province, Nepal, including wealth, education, age, residence, occupation, caste/ethnicity, religion, and media exposure. These have been presented in Table 1. Socio-economically, women in the middle and poorer wealth quintiles reported higher fertility intentions (15.6% each) compared to those in the richest quintile (6.0%). Education was a significant determinant: women with primary education (20.2%) and secondary education (18.8%) expressed higher fertility intentions compared to those with no formal education. (14.5%) or tertiary education (1.3%). A similar trend was observed for partner’s education: partners with secondary education were associated with higher fertility intentions (15.5%) than those with no formal education (7.8%).

Table 3 also presents that demographically, fertility intentions were highest among younger women, especially those aged 15–19 (26.5%) and 20–24 age group. Fertility intentions declined significantly with increasing age, nearly disappearing among women aged 40-44and 45–49 years. Parity had a significant negative relationship with fertility intention. Only one-third of the women had no children (33.8%), and this group had the highest ambition of having more children (33.2%). Women having 3–4 children (23.0%), and those having 5 or above children (3.9%), on the other hand, had little intention to have further children (2.6% and 0.2%, respectively). The negative correlation of the parity levels shows that more parity is closely linked with the completed fertility. (Table 1). Urban women expressed slightly higher fertility preferences (41.7%) than rural women (13.1%). Women’s employment status also influenced fertility intentions, with non-working women (29.2%) and those employed in agriculture (18.1%) expressing a stronger desire for more children. Caste/ethnicity and religion were also significant. Women from other Terai castes (26.1%) and Terai Dalits (11.1%) reported higher fertility intentions, as did Muslim women (9.7%). Media exposure was also associated with fertility intentions; women who regularly accessed newspapers, radio, and television expressed stronger fertility desires than those with no media exposure. Women who watched television at least once a week showed a relatively higher fertility intentions (19.3%) than those who had never watched television (23.9%). These findings demonstrate the complex interplay of economic status, access to information, and social-cultural identity in shaping reproductive aspirations within Madhesh Province.

**Table 3:** Fertility preferences across women’s socio-economic status and their demographic characteristics

Variables	Number(N)	Percent (%)	Fertility preferences	
			**No (%)**	**Yes (%)**
Socio-economic variables				
Wealth quintile				
Poorest	125	8.8	3.8	5.0
Poorer	422	25.7	10.2	15.6
Middle	531	29.7	14.1	15.6
Richer	398	23.7	11.0	12.7
Richest	167	12.1	6.1	6.0
Level of education				
No education	568	35.8	21.3	14.5
Primary	548	32.3	12.1	20.2
Secondary	491	29.7	10.9	18.8
Higher	36	2.2	0.9	1.3
Partner’s level of education				
No education	243	20.7	12.9	7.8
Basic	425	32.9	18.7	14.2
Secondary	424	34.7	19.2	15.5
Higher	84	6.6	3.9	2.7
Highest	61	5.2	2.6	2.6
Demographic variables				
Age				
15–19	462	27.3	0.8	26.5
20–24	436	27.3	8.5	18.8
25–29	276	17.1	11.2	5.9
30–34	189	11.6	9.1	2.5
35–39	136	8.2	7.5	0.8
40–44	96	5.7	5.4	0.3
45–49	48	2.7	2.7	
Parity				
No children	557	33.8	0.6	33.2
1–2 children	653	39.4	20.7	18.7
3–4 children	371	23.0	20.3	2.6
5 + children	62	3.9	3.7	0.2
Residence				
Urban	947	74.4	32.7	41.7
Rural	696	25.6	12.5	13.1
Occupation				
Not working last 12 months	716	45.8	16.6	29.2
Professional/technical	52	3.2	1.2	2.0
Clerical	9	0.8	0.1	0.6
Sales and service	68	4.3	2.6	1.7
Skilled manual	70	4.8	2.2	2.6
Unskilled manual	30	1.7	1.1	0.5
Agriculture	698	39.5	21.4	18.1
Other				
Caste/Ethnicity				
Hill Brahmin	20	1.8	1.2	0.6
Hill chhetri	12	1.0	0.6	0.4
Terai brahmin/chhetri	46	3.1	1.4	1.7
Other terai caste	767	47.7	21.6	26.1
Hill dalit	9	0.6	0.3	0.3
Terai dalit	333	18.1	7.0	11.1
Newar	22	0.8	0.4	0.4
Hill janajati	45	2.9	1.8	1.1
Terai janajati	131	6.5	3.3	3.3
Muslim	256	17.4	7.7	9.7
Other	2	0.1		0.1
Religion				
Hindu	1,355	81.0	36.7	44.3
Buddhist	28	1.2	0.7	0.5
Muslim	255	17.3	7.6	9.7
Kirat	3	0.4	0.1	0.2
Christian	2	0.1	0.1	0.1
Frequency of reading newspaper or magazine				
Not at all	1,476	89.3	41.0	48.3
Less than once a week	114	7.4	3.3	4.1
At least once a week	53	3.3	0.9	2.4
Frequency of listening to radio				
Not at all	1,081	66.5	30.2	36.3
Less than once a week	375	22.3	10.1	12.2
At least once a week	187	11.2	4.9	6.3
Frequency of watching television				
Not at all	714	44.6	20.7	23.9
Less than once a week	344	20.5	8.9	11.6
At least once a week	585	34.9	15.6	19.3

Source: Computed from NDHS 2022

Table 4 presents the results of both unadjusted (Model 1) and adjusted (Model 2) logistic regression analyses identifying factors associated with fertility intentions among women of reproductive age in Nepal. The unadjusted analysis revealed significant associations with several socio-economic variables. For example, belonging to the "poorer" wealth quintile increased the odds of fertility intentions (cOR = 1.56, 95% CI: 1.15–2.13) compared to the richest quintile. However, this association lost significance after adjustment (aOR = 1.16, 95% CI: 0.67–2.02). Likewise, women with no education (cOR = 0.47, 95% CI: 0.25–0.86) and partners with no education (cOR = 0.60, 95% CI: 0.36–0.99) showed significantly lower fertility intentions in the unadjusted model, but these effects became non-significant after accounting for confounding factors.

**Table 4:** Socioeconomic and demographic factors associated with fertility preferences among women of reproductive age in Nepal

**Variables**	Model 1 cOR 95% CI	Model 2 aOR 95% CI
Socio-economic variables		
Wealth quintile		
Poorest	1.34 (0.91–1.98)	1.29 (0.66–2.54)
Poorer	1.56 (1.15–2.13)***	1.16 (0.67–2.02)
Middle	1.12 (0.83–1.51)	1.05 (0.63–1.74)
Richer	1.17 (0.86–1.59)	1.11 (0.69–1.80)
Richest	Ref	Ref
Level of education		
No education	0.47 (0.25–0.86)**	1.11 (0.35–3.51)
Primary	1.15 (0.62–2.13)	0.93 (0.30–2.88)
Secondary	1.19 (0.64–2.21)	0.88 (0.30–2.62)
Higher	Ref	Ref
Partner’s level of education		
No education	0.60 (0.36–0.99)**	0.77 (0.41–1.45)
Basic	0.76 (0.47–1.22)	0.95 (0.52–1.75)
Secondary	0.81 (0.50–1.30)	1.21 (0.65–2.25)
Higher	0.68 (0.38–1.24)	1.33 (0.58–3.04)
Highest	Ref	Ref
Demographic variables		
Age		
15–19	Ref	Ref
20–24	0.07 (0.04–0.12)***	0.14 (0.08–0.25)***
25–29	0.02 (0.01–0.03)***	0.03 (0.02–0.06)***
30–34	0.01 (0.01–0.02)***	0.02 (0.01–0.03)***
35–39	0.00 (0.00–0.01)***	0.01 (0.00–0.02)***
40–44	0.00 (0.00–0.01)***	0.00 (0.00–0.01)***
45–49	0 (0–0.001) ***	—
Parity		
No children	Ref	Ref
1–2 children	23.898(8.389–68.078) ***	18.205(5.579–59.404) ***
3–4 children	3.589(1.269–10.151) **	2.808(0.902–8.739) *
5 + children	0.544 (0.162–1.821)	0.575(0.162–2.041)
Residence		
Urban	Ref	Ref
Rural	0.82 (0.67–1.00)*	0.67 (0.49–0.92)**
Occupation		
Not working	2.77 (1.76–4.36)***	1.27 (0.59–2.72)
Professional/technical	2.78 (1.43–5.42)***	2.48 (0.83–7.45)
Skilled manual	1.87 (1.04–3.38)**	0.80 (0.31–2.05)
Unskilled manual	0.78 (0.33–1.80)	1.07 (0.27–4.28)
Agriculture	1.33 (0.84–2.09)	1.21 (0.56–2.58)
Sales/service (Ref)	Ref	Ref
Caste/Ethnicity		
Hill Brahmin (Ref)	Ref	Ref
Hill Chhetri	1.13 (0.36–3.56)	3.05 (0.58–15.92)
Terai Brahmin/Chhetri	2.29 (0.97–5.39)*	2.17 (0.57–8.26)
Other Terai Caste	2.36 (1.16–4.79)**	1.37 (0.46–4.09)
Hill Dalit	1.64 (0.45–6.01)	2.98 (0.46–19.22)
Terai Dalit	3.09 (1.49–6.39)***	1.55 (0.49–4.93)
Newar	2.29 (0.68–7.69)	4.54 (0.87–23.70)*
Hill Janajati	1.24 (0.52–2.97)	0.39 (0.07–2.07)
Terai Janajati	1.97 (0.91–4.28)*	1.77 (0.54–5.82)
Muslim	2.43 (1.17–5.03)**	0.00 (0.00–NA)
Religion		
Hindu	Ref	Ref
Buddhist	0.56 (0.25–1.26)	6.21 (1.00–38.39)**
Muslim	1.05 (0.83–1.32)	547,308 (0.00–NA)
Kirat	1.25 (0.28–5.59)	8.67 (0.51–148.28)
Christian	1.20 (0.11–13.33)	-
Frequency of reading newspaper or magazine		
Not at all	0.47 (0.27–0.81)***	0.49 (0.21–1.16)
Less than once a week	0.49 (0.27–0.92)**	0.38 (0.15–0.99)**
At least once a week	Ref	Ref
Frequency of listening to radio		
Not at all	0.94 (0.70–1.25)	1.34 (0.83–2.16)
Less than once a week	0.95 (0.68–1.31)	1.42 (0.84–2.41)
At least once a week	Ref	Ref
Frequency of watching television		
Not at all	0.93 (0.76–1.14)	1.20 (0.86–1.69)
Less than once a week	1.05 (0.82–1.34)	1.17 (0.79–1.71)
At least once a week	Ref	Ref
N	1643	1192
Pseudo***R***^2^	0–0.378	0.285

Exponential coefficients presented; 95% confidence intervals in brackets

*cOR* Crude Odds Ratio, *aOR* Adjusted Odds Ratio

*Significance levels:*

^*^p < 0.05

^**^p < 0.01

^***^p < 0.001

Age came out as a major determinant in both the models. Fertility intentions decreased progressively with age relative to 15–19-year reference group, reaching near-zero probability (aOR ≈ 0.00) among women aged 40–44 years. Rural residence was associated with lower odds of fertility intentions compared to women living in urban areas (aOR = 0.67, 95% CI: 0.49–0.92).

The parity was significantly and gradual associated with fertility intention. The women with 1–2 children were significantly more likely to have further birth intentions as compared to those with none (aOR = 18.21, 95 percent CI = 5.58 59.40, *p* = 0.001). The magnitude of the association decreased with the increased parity-women with 3–4 children had a modest relationship (aOR = 2.81, 95% CI = 0.90874), women with more than 5 children had no significant association (aOR = 0.58, 95% CI = 0.16204). This trend indicates that fertility intention is decreasing as the number of children is increasing (Table 2). Occupational status was significant in the unadjusted model; for example, women in professional/technical occupations had substantially higher odds (cOR = 2.78). However, these associations attenuated after adjustment. Findings for ethnicity and religion were mixed; Terai Dalit (cOR = 3.09), Muslim (cOR = 2.43), and Newar women (aOR = 4.54) showed higher fertility preferences initially, but these associations became statistically insignificant after adjustment, implying that their effects are mediated by other factors. Media exposure showed a protective effect; women who read newspapers less than once a week had lesser odds of fertility intentions (aOR = 0.38, 95% CI: 0.15–0.99) compared to weekly readers. In summary, key predictors of fertility intentions identified in this study include younger age, rural residence and media exposure. Wealth status, education, and religious affiliation did not retain significant independent associations after adjustment for confounders.

## Discussion

This study examined the socio-economic and demographic determinants of fertility intentions among women of reproductive age in Madhesh Province using the data derived from the 2022 Nepal Demographic and Health Survey [6]. After adjusting for major covariates (age, parity, education and residence); age, parity and media exposure remained the most significant predictors of fertility intentions. In contrast, the effects of wealth, education, caste and religion became statistically insignificant after adjustment. These results highlight the complex and context-specific nature of reproductive decision making in Madhesh province, consistent with theoretical expectations of fertility behavior [8, 27].

Age emerged as the strongest determinants. Younger women particularly those aged 15–24 years and with fewer children, were substantially more likely to express desire for additional children. This pattern aligns with evidence from South Asia and sub-Saharan Africa, where younger women’s fertility intentions reflect incomplete family formation and sociocultural norms that favor larger families early in reproductive life [8, 26]. Fertility intentions declined sharply with increasing age likely due to both biological constraints and societal expectations that discourage childbearing at older ages [28].

Place of residence also showed an interesting pattern. Contrary to earlier propositions that rural women have higher fertility desires [27], the adjusted results indicated that rural women in Madhesh were less likely to want more children than urban women. This reversal may reflect recent urbanization trends, improved access to education and family planning information in rural areas, and migration of higher fertility households to urban centers. It also may suggest that urban women, influenced by social expectations of larger families or differing life-stage structures are more likely to plan additional births. These findings should, however be interpreted cautiously, as unmeasured factors such as migration or employment could also explain the variation [6, 29]. Education, wealth status and occupation were significantly associated with fertility intentions in the unadjusted analysis, but these relationships became non-significant once confounding factors were controlled for. This suggests that their effects may be mediated by variables such as age, and access to information rather than acting independently. Evidence from the other South Asian regions similarly indicated that reproductive autonomy and exposure to information often have stronger predictive power than formal education in shaping fertility behavior [30, 31]. The disappearance of occupational effects after adjustment implies that employment alone does not determine fertility preferences without considering the wider socio-cultural context [32].

Caste, ethnicity and religion produced mixed results. While Terai Dalits, Muslims, and Newars initially appeared more likely to intend further childbearing, these differences lost statistical significance after adjustment. Contrarily, Buddhist women showed higher odds in the adjusted model though the sample size warrants cautious interpretation. This pattern suggests that caste and religion influence fertility more indirectly through disparities in education, access to health services and gender norms rather than as standalone cultural determinants [33, 34].

Media exposure, especially newspaper reading, was inversely associated with fertility intentions. Women who reported reading newspapers less than weekly had lower odds of intending more children than regular readers (aOR = 0.38, 95% CI: 0.15–0.99), suggesting that exposure to reproductive health information through print media may promote smaller family. The weaker associations found for radio and television may reflect content gaps, accessibility differences or varying audience engagement. Comparable findings from other low and middle-income countries show that print media often conveys more targeted reproductive health messages than radio or television [35–37].

These results indicate that fertility intentions in Madhesh are driven more by lifestage and informational factors than by structural variables such as education or wealth status. The findings emphasize the importance of designing media-based, youth-focused and rural-sensitive reproductive health programs and of addressing underlying gender norms that continue to shape fertility decisions among the marginalized groups [38].

A key strength of this study is the use of most recent, nationally representative NDHS 2022 dataset, enhancing the reliability and generalizability of the findings for Madhesh Province. The large sample size and use of both unadjusted and adjusted regression models strengthen the analytical rigor and help minimize bias. However, some limitations should be acknowledged. The cross-sectional design prevents drawing causal conclusions, and self-reported fertility intentions may be influenced by recall or social desirability bias, especially among older and women with low education. In addition, small subgroup sizes for certain religious minorities limited the statistical precision, resulting in wider confidence intervals [39].

## Conclusion

This study provides a population-based understanding of fertility intentions among women of reproductive age in Madhesh Province, Nepal, using data from the 2022 Nepal Demographic Health Survey (NDHS). The findings reported age, residence and media exposure as the most significant determinants of fertility intentions. Although wealth, education, and caste/ethnicity initially appeared influential, their effects diminished after adjusting for confounding factors.

Younger women, those with one to two children, urban residents, and women with access to print media were more likely to express fertility intentions, suggesting that reproductive decision-making in Madhesh is shaped by both life-stage and informational factors rather than by structural or economic status alone.

To align fertility intentions with positive reproductive health outcomes, targeted awareness initiatives particularly through accessible and culturally relevant print media should be strengthened. Policies should focus to reduce the urban–rural disparities in family planning access and address persistent social and gender norms influencing fertility behavior. Provincial health authorities are encouraged to develop youth-centered, equity-focused reproductive health strategies that empower women, improve access to contraception and foster informed reproductive choices across diverse communities in Madhesh Province.

## Data Availability

No datasets were generated or analysed during the current study.
